# From Data Mining of *Chitinophaga* sp. Genome to Enzyme Discovery of a Hyperthermophilic Metallocarboxypeptidase

**DOI:** 10.3390/microorganisms9020393

**Published:** 2021-02-14

**Authors:** Gabriela Cabral Fernandes, Elwi Guillermo Machado Sierra, Paul Brear, Mariana Rangel Pereira, Eliana G. M. Lemos

**Affiliations:** 1 Department of Technology, São Paulo State University (UNESP), Jaboticabal, São Paulo State 14884-900, Brazil; gfernandes1403@gmail.com (G.C.F.); elwi.machado@unisimonbolivar.edu.co (E.G.M.S.); 2 Graduate Program in Agricultural and Livestock Microbiology, São Paulo State University (UNESP), School of Agricultural and Veterinarian Sciences, Jaboticabal, São Paulo State 14884-900, Brazil; 3 Laboratorio de Investigación en Microbiología, Facultad de Ciencias Básicas y Biomédicas, Universidad Simón Bolívar, Barranquilla 080002, Colombia; 4 Department of Biochemistry, University of Cambridge, Cambridge CB21GA, UK; pdb47@cam.ac.uk; 5 CAPES Foundation, Ministry of Education of Brazil, Brasília-DF 70.040-02, Brazil

**Keywords:** metallocarboxypeptidase, M32 family of peptidases, *Chitinophaga* sp.

## Abstract

For several centuries, microorganisms and enzymes have been used for many different applications. Although many enzymes with industrial applications have already been reported, different screening technologies, methods and approaches are constantly being developed in order to allow the identification of enzymes with even more interesting applications. In our work, we have performed data mining on the *Chitinophaga* sp. genome, a gram-negative bacterium isolated from a bacterial consortium of sugarcane bagasse isolated from an ethanol plant. The analysis of 8 Mb allowed the identification of the *chtcp* gene, previously annotated as putative Cht4039. The corresponding codified enzyme, denominated as ChtCP, showed the HEXXH conserved motif of family M32 from thermostable carboxypeptidases. After expression in *E. coli*, the recombinant enzyme was characterized biochemically. ChtCP showed the highest activity versus benziloxicarbonil Ala-Trp at pH 7.5, suggesting a preference for hydrophobic substrates. Surprisingly, the highest activity of ChtCP observed was between 55 °C and 75 °C, and 62% activity was still displayed at 100 °C. We observed that Ca^2+^, Ba^2+^, Mn^2+^ and Mg^2+^ ions had a positive effect on the activity of ChtCP, and an increase of 30 °C in the melting temperature was observed in the presence of Co^2+^. These features together with the structure of ChtCP at 1.2 Å highlight the relevance of ChtCP for further biotechnological applications.

## 1. Introduction

Extremophilic microorganisms are widely distributed around the world. Their defining characteristic is the ability to survive under extreme conditions such as low and high temperatures (psychrophiles and thermophiles from −2 °C to 20 °C and 60 to 115 °C, respectively), different salt concentrations (halophiles are able to survive in the presence of 2 M to 5 M of NaCl), acid levels and basic pH levels (acidophilic and alkaliphilic microorganisms are alive in pH < 4 and pH > 9, respectively). To survive in these environments, microorganisms have developed stable enzymes with effective activity, even under these extreme conditions. Over the last few years, many of these microorganisms have attracted enormous interest for a variety of biotechnological applications across different sectors such as the agricultural, energy, environmental, food, health, pharmaceutical industries and textile sectors [1]. Currently, more than 500 industrial products are being produced using enzymes, and 65% of these products are in industrial applications such as detergents, starches, leather, textiles, personal care products, pulp and paper, 25% are used in food processing and 10% are used in food supplements for animals [2]. The demand for industrial enzymes is constantly growing and the estimated market in 2018 was $7100 million. This value is currently increasing by 8% per year, driven mainly by an increasing need for sustainable solutions [3]. Nevertheless, many industrial processes still use chemical synthesis, which generally presents low catalytic efficiency, a lack of enantiomeric specificity for chiral synthesis and also requires extreme conditions (e.g., high temperatures, low pH and high pressure). In addition, the use of organic solvents leads to polluting waste. In view of this, enzymes are very useful biotechnological applications, as they work under atmospheric conditions (pH, temperature and pressure), have a long half-life, have high regio- and enatio-selectivity (therefore inefficient protecting group and chiral synthesis strategies are not needed) and can accelerate the reaction by up to 10^8^ fold [3]. However, some of the disadvantages of using enzymes are the production prices, freshwater consumption, frequent microbial contamination, discontinuous production processes and the efforts required for product separation and purification [4,5,6,7].

Given the points mentioned above, the importance of basic research into finding more versatile enzymes is clear. The analysis of complete genomes is a useful strategy that can successfully be used for the discovery of novel enzymes or metabolites for industrial purposes [8]. Among the enzymes which have been discovered with this approach are endoglucanases, laccases, nitrilases, reductases and xylanases [9]. This is in addition to other enzymes of major industrial value such as peptidases [10], lipases [11,12] and ß-glucosidases [13]. This approach shows great potential for enzyme discovery from microorganisms. For example, the genome analysis of *Chitinophaga pinensis* revealed a wide catabolic machinery including the discovery of 169 genes encoding for glucosidase enzymes belonging to 49 different families [14], 213 putative or non-putative genes and 74 homologous genes encoding for proteases which have not been characterized [15]. The large number of genes encoding for enzymes makes *Chitinophaga* bacteria a microorganism with a high biotechnological potential.

The genus Chitinophaga was first described in the 1980s and is characterized as a gram-negative bacteria, positive oxidase and non-pathogenic and mesophilic characteristics [16], with a filamentous morphology that transforms when aging into spherical bodies (mixospores), without the production of a fruiting body [17]. The genus stands out for participating actively in soils [18,19,20], specifically in the rhizosphere [21], arsenic-contaminated soil [22] and vermicompost [23], where the biomass of plants and fungi is intensely degraded. This degradation is extremely significant for carbon recycling in the natural environment [24].

This genus comprises 31 species [25] and a very diverse carbohydrate degradation metabolism [18,24]. One of the main reasons for interest in this genus is its ability to hydrolyze chitin and in some species the ability to even hydrolyze cellobiosis. Most studies of the genus *Chitinophaga* are centered on phylogeny and taxonomy, and just a few groups have addressed the study of the genus from a functional and metabolic point of view with an industrial application in mind. The first approach of this type was carried out by Nawrath et al. (2010) [26] through the synthesis and characterization of 2-methyltetrahydrothiophen-3-one, which is used extensively in food production; another example is the isolation of antibiotics such as p-quinone and Elansolido A [27], and more recently the production of diterpene synthase [28] and β-glucan [29]. However, it should be noted that not much is known about the proteolytic machinery of this bacterial genus.

Many industrial biotechnology applications of proteases are reported in the literature. One of these is the use of controlled proteolysis of target proteins. This is achieved through the use of proteases to produce protein hydrolysates with bioactivity and desirable flavors. This strategy has become an important and sustainable approach, and has the aim of providing food with improved properties [30]. Enzyme discovery of high-efficiency proteases showing attractive features, such as thermostability, are relevant to industry in order to reduce costs. There are several commercial proteases, most of which are forms of endoprotease, except for some which appear as a mixture of endoproteases and exoproteases, such as Flavorourme. Exoproteases hydrolyze the ends of the peptide chain (N or C terminus of a protein) by removing a single amino acid or, sometimes, two or three peptides. These enzymes play important roles in improving hydrolysis efficiency as part of the pre-processing steps, in addition to physical methods [31], and in modifying the taste of protein-rich products [30].

Metallocarboxypeptidase (EC 3.4.17.19) are peptidases that present a conserved HEXXH or HXXEX catalytic motif that binds a metal ion [32,33]. This type of protease exhibits a relatively broad specificity for hydrophilic (neutral and basic) and hydrophobic (aliphatic and aromatic) residues in the C-terminal, and shows high activity in the temperature range of 75 °C to 80 °C and in the pH range of 6.8 to 7.2 [34]. Currently, metallocarboxypeptidases have been isolated from hemophilic microorganisms such as *Thermus aquaticus* [35], *Thermococcus* sp. [36], *Pirococcus furiosus* [37], *Thermus thermophilus* [34,38], *Geobacillus* SBS-4S [33], *Fervidobacterium islandicum* [32] and *Bacillus subtilis* [39,40]. The main application of these proteases is in cheese ripening [41] and in the degradation of keratin-rich residues [32].

In a previous work [19], the complete genome of a *Chitinophaga* sp. isolated from a bacterial consortium of sugarcane bagasse was sequenced and 7173 open reading frames (ORF) were identified, while 72 were annotated as potential genes for the coding of proteolytic enzymes. However, to date despite their great potential, none of these genes have been biochemically characterized. This work is the first study of these enzymes. Here, we moved from the selection of a gene coding for a putative proteolytic enzyme to functional and structural characterization of the protein encoded by it. The selected gene, denominated *chtcp*, shares 40% identity with the thermophilic carboxypeptidase FisCP from *Fervidobacterium islandicum* and TthCP from *Thermus thermophilus*. The *chtcp* gene was cloned into an expression vector and the recombinant protein was expressed in a soluble form in *E. coli* for functional and crystallography studies. ChtCP showed *k*_cat_/k_M_ (s^−1^·M^−1^) of 2.48 × 10^2^ against benzyloxycarbonyl Ala-Trp and was active over a wide pH range. The melting temperature (Tm) of ChtCP was obtained at different pH levels and in the absence of metals, the unfolding occurred at approximately 62 °C. Surprisingly, in the presence of Co^2+^ or Cd^2+^, an increase in 25 °C and 17 °C in Tm was observed. Our sequence and structural analysis showed the alpha-beta folding of this protein with the conserved HEXXH zinc-binding motif and the active site situated in a deep substrate binding cleft. With the functional and structural studies of ChtCP, we hope to provide a reliable dataset for all enzymes sharing the same sequence or structural features. This is the first work showing the biochemical properties of a metalloenzyme obtained from this particular *Chitinophaga* sp. isolated from sugarcane bagasse. We propose that the high temperatures tolerated by ChtCP will widen this enzyme’s potential industrial applications. This will be particularly advantageous in processes such as fermentation in the food industry and in improving dyeing quality and gloss enhancement in the textile industry to prevent shrinkage and decrease felting.

## 2. Materials and Methods

### 2.1. DNA Sequencing of Chitinophaga sp. Genome

The microorganism *Chitinophaga* sp. CB10 was isolated from a bacterial consortium taken from a soil with high rate of biomass degradation and cultivated in sugarcane bagasse. This type of bacteria was isolated in the Biochemistry Laboratory of Microorganisms and Plants (LBMP) localized at São Paulo State University, Câmpus of Jaboticabal. The complete genome of *Chitinophaga* sp. CB10 was sequenced by Ion Torrent platform (Thermo Fisher Scientific, Waltham, MA, USA) [19], and the ORF prediction and annotation was performed in RAST (Rapid Annotation of microbial genomes using Subsystem Technology) [42]. *Chitinophaga* sp. CB10 genome was deposited in the NCBI GenBank under the accession number MLAV00000000.1.

### 2.2. Selection of Potentially Interesting Proteases Via Data Mining

A selection criteria was used to identify genes encoding potentially interesting proteolytic enzymes: (1) E-value < 1 × 10^−4^, a relative strict threshold to guarantee a high coverage and low false-positive results; (2) sequence identity ≤ 40% with ORFs already described and characterized in the literature; (3) the position of the residues from the catalytic site and metal binding; (4) the presence of conserved motifs which define the peptidases; and (5) sequence alignment coverage of ≤ 60%. All redundant strings with values greater than 90% were excluded.

We tried to focus our research on enzymes with lower sequence identity compared to those already described in the literature. Thus, the sequence of potential candidates were validated by comparing them against databases such as BRENDA [43], UniProt [44] and MEROPS [15]. The proteins of interest were selected taking into account the physicochemical characteristics of homologous proteins, which were previously discussed and characterized in the literature. The following parameters were considered: the optimum pH 5 ≤ or ≥ 8, optimum temperature of 10 °C ≤ or ≥ 50 °C and the stability in the presence of denaturing agents or organic solvents, which are desired enzymatic characteristics in the industry.

### 2.3. Sequence Analysis of Gene Chtcp

The conserved motifs and protein signatures were analysed in InterProScan [45], while the theoretical physico-chemical and melting temperature of the protein were generated by ProtParam ExPASy [46] and Tm Predictor [47], respectively. To estimate the evolutionary conservation of each translated amino acid, the CLUSTALW program [48] and the ConSurf server [49] were used. A phylogenetic tree was built to infer the evolutionary history of ChtCP and for that, 38 sequences of members representing M32 family were retrieved from the MEROPS database and together with ChtCP sequence, the sequences were aligned by the CLUSTALW program [48]. The output file obtained was used as an input file to generate the tree in MEGA7 [50] via the Maximum Likelihood method, based on the JTT matrix-based model [51].

### 2.4. Cloning of Gene Chtcp

The cloning of *chtcp* gene (GenBank accession number gene: BK014293) into an pHAT2 expression vector [52] was performed following the protocols described in Pereira et al., 2015 and Maester et al., 2016 [11,12], and by using the forward and reverse primers *CBXF1f* TATATCCATGGCAGGAAGCAAATCTACAGCAG and *CBXF2r* TATATAAGCTTATCCCCGGAGGAACTTGCCTGC, respectively.

### 2.5. Expression, Extraction and Purification of Recombinant ChtCP

For the expression of the ChtCP recombinant protein, an isolated colony of the pHAT2-*chtcp* construct, previously transformed into *E. coli* BL21 (DE3), was inoculated in 500 mL of LB medium containing 50 μg·mL^−1^ ampicillin. The culture was incubated at 37 °C and 200 rpm until the logarithmic growth phase was reached and at optical density (OD), 600 nm of the culture was induced with 0.4 mM of IPTG for 2 h at 37 °C. After expression, cells were collected by centrifugation at 6.387× *g* for 20 min, followed by resuspension of the cells with a 10 mL extraction buffer (100 mM tris-HCl pH 8.0, 200 mM NaCl; 0.2% triton X100 and 10 mM imidazole) per liter of culture. The cells were lysed by using a Branson Sonifier 250 sonicator (Thermo Fisher Scientific, Waltham, MA, USA) with a 30% cyclic ratio and 30 s intervals (Duty cycle 20; Out put 5) for 30 min. After lysis, the samples were spun down at 16.266× *g*, 4 °C for 20 min. The soluble extract of the ChtCP protein was purified by affinity chromatography using Ni-NTA agarose resin (Qiagen, Venlo, Limburg, The Netherlands) and eluted in a continuous gradient of imidazole; at the 10 to 500 mM stage, the quality of the eluted fractions was investigated by denaturing electrophoresis on SDS-PAGE polyacrylamide gel [53]. The protein concentration was determined using spectrophotometric analysis and by applying the theoretical extinction coefficient (89,855 M^−1^·cm^−1^), and was also determined via the Bradford method [54] with bovine serum albumin as a reference.

After nickel column purification, an additional step of purification was included and the approximate molecular weight of the recombinant ChtCP protein could be also determined via gel filtration. The purified ChtCP was injected into a loop of 5 mL and later eluted in a HiLoad Superdex 200 16/60 column (GE Healthecare, Little Chalfon, UK), previously equilibrated in buffer A [Tris-HCl 20 mM pH 8; 200 mM de NaCl and 0.5% Glycerol]. The run was performed in a cold room (4 °C) by using a flow of 1 mL/min, with the fractions collected in a 96-well plate. The absorbance was monitored at 280 nm by an Akta Pure System (GE Healthecare, Little Chalfon, UK) and the chromatogram obtained was used to compare with the standard (Sigma-Aldrich, St. Louis, MO, USA), which was also purified under the same conditions mentioned above. The elution volume of ChtCP was compared to each standard protein [bovine thyroglobulin (670 kDa), γ-globulin (150 kDa), albumin (43 kDa), ribonuclease A (13.7 kDa) and ρ-amino benzoic acid (pABA, 0.13 kDa)]. The elution volume value of ChtCP and each standard was used for linear regression analysis and the data was processed on Microsoft Excel, version 3.04 (Microsoft Corporation, Redmond, WA, USA).

### 2.6. Enzyme Activity and Kinetic Parameters of ChtCP

The proteolytic activity of the recombinant ChtCP protein was evaluated following the protocol proposed by Doi et al., 1981 [55]. To detect the enzymatic activity of ChtCP, 0.41 to 1.6 µM of the enzyme was added to 2 mM of benzyloxycarbonyl Ala-Arg, and the reaction incubated at 20 °C for 30 min. The reaction was stopped by addition of 0.2 mL of Cd-ninhydrin followed by an incubation step at 80 °C for 5 min and cooling on ice for 5 min. The reactions were carried out in triplicate in 50 mM hepes pH 8.0 in a final volume of 100 µL. The absorbance was measured at 500 nm by spectro-photometrical quantification in SpectraMax^®^ M3 Microplate Reader (BioTek Instruments, Winooski, VT, USA), with an unheated assay mixture containing Cd-ninhydrin reagent serving as a blank. Absorbances were compared with an alanine, arginine, asparagine and tryptophan standard curve in order to calculate specific activities. One unit of ChtCP was defined as the amount of enzyme necessary to produce 1 µmol of product per minute under the conditions tested [56]. To determine the kinetic parameters of ChtCP, the assay was performed by varying the concentrations of benzyloxycarbonyl Ala-Arg (ZAR), benzyloxycarbonyl Ala-Ala (ZAA), benzyloxycarbonyl Ala-Trp (ZAW) or benzyloxycarbonyl Ala- Asn (ZAN) from 0.195 to 25 mM. These substrates were diluted in methanol to achieve the concentration of 100 mM and were further diluted in 50 mM hepes pH 8.0, where the reaction occurred at 65 °C, in the presence and absence of cofactors. The assays were carried out in triplicate and a negative control was performed for each condition tested, with no ChtCP in order to measure the spontaneous hydrolysis of the substrates. Analysis of the linear regressions was performed in Microsoft Excel, version 15.27, to determine the initial speed (Vo) of the reaction. The kinetic parameters V_max_ and K_M_ were calculated using Kaleidagraph [57] by non-linear lest square fits of initial rates to the Michaelis–Menten equation and the catalytic rate constant *k*_cat_ (s^−1^) was calculated from the initial steady-state velocity according to the equation *k*_cat_ = V_max_/[E].

### 2.7. The Effect of Temperature, pH and Cofactors on ChtCP Activity

To evaluate the effect of pH change on the ChtCP activity, 100 mM of the following buffers were tested: sodium citrate (pH 4.0, 4.5, 5.0, 5.5 and 6.0); tris-HCl (pH 7.0, 7.5, 8.0 and 8.5); sodium bicarbonate (pH 9.0, 9.5 and 10.0).

The effect of temperature on ChtCP activity was also investigated. A wide temperature range was tested, from 20 to 99 °C. ChtCP at 1 mg/mL was incubated for 1 h at the corresponding temperatures, and the residual activity of the enzyme was measured after incubation with 2 mM ZAR. All reactions were carried out in 100 mM Tris-HCl pH 8.0 in triplicate and an enzyme-free blank was performed to subtract auto-hydrolysis.

The effect of the presence of selected ions on the enzymatic activity of ChtCP was also investigated. Next, 2 mM of the following ions was added to the reactions: CdCl_2_, CoCl_2_, CrSO_4_, MgSO_4_, MnSO_4_, ZnCl_2,_ CuSO_4_, BaCl_2_, MgCl_2_, NiCl_2_, FeCl_3_ and CaCl_2_. For this assay, 0.5 μM of the enzyme was tested in the presence of the substrate ZAR and 100 mM Tris-HCl pH 8.0. The reaction was incubated for 5 min and monitored for 30 min.

### 2.8. Determination of Tm Via Differential Scanning Fluorometry (DSC)

Differential scanning fluorometry was performed in order to determine the melting temperature of ChtCP in the presence and absence of metal ions. Next, 0.5 μM of SYPRO Orange (Sigma-Aldrich, St. Louis, MO, USA) was added to 4 μg of purified ChtCP (treated and not treated with EDTA), and the enzyme was tested against 0.5 to 10 mM of different metals ions (CdCl_2_, CoCl_2_, CrSO_4_, MgSO_4_, MnSO_4_, ZnSO_4_). The assay occurred in the presence of 50 mM of hepes and a pH of 8 in a 96-well, and with a final volume of 20 μL. The plate was sealed and equilibrated at 25 °C for 10 min followed by a constant increase of 0.5 °C per minute from 25 to 95 °C. The released fluorescence was monitored by the qPCR Stratagene Mx 3000P (Agilent, Santa Clara, California, USA) system and the melting point (Tm) determined by fitting the data on Boltzmann sigmoidal with GraphPad Prism 5 (GraphPad Software, La Jolla, CA, USA). The points measured before and after the minimum and maximum fluorescence intensity, respectively, were excluded from the analysis [58,59]. The T*_m_* values in the absence of metal ions were measured by melting curve analysis on a Rotor-Gene^TM^ 6000 real-time analyzer (Corbett Research, Mortlake, Australia) in 50 mM Tris-HCl buffer in different pH levels (7.0, 8.0 and 9.0).

### 2.9. Crystallisation of ChtCP

The purified recombinant protein ChtCP at a final concentration of 12 mg/mL in buffer A was submitted to initial crystallization trials using the following commercial kits: JCSG suite (Qiagen, Hilden, Germany), Wizard III and IV (Emerald BioSystems, Bainbridge Island, Washington, DC, USA) and Classics (Jena Bioscience, Jena, Germany). The first crystals were obtained in 0.1 M trisodium citrate pH 5.0; 220% PEG 6K (commercial kit JCSG, well F7), and were further optimized by changing salt and protein concentration. For the optimization, plates were manually prepared through a vapor-diffusion technique using 1 µL:1 µL and 1 µL:0.5 µL of mother liquor and protein, respectively. All crystallization plates were incubated at 21 °C.

### 2.10. Data Collection and Structure Determination of ChtCP

X-ray diffraction data were collected at the Diamond Light Source on the beamline IO4 and data from automated data processing with autoProc [60] were used for structure determination. The phases were solved by molecular replacement in Phaser [61] using the model PDB: 5GIV and iteratively rebuilt and refined with refmac [62] and coot [63]. The coordinates have been deposited to the Protein Data Bank under accession number 7A03. Data collection and refinement statistics are shown in Supplementary Table S3.

## 3. Results

### 3.1. From Prediction of Peptidases in the Chitinophaga sp. Genome to Sequence Analysis of ChtCP

**Figure 1:**
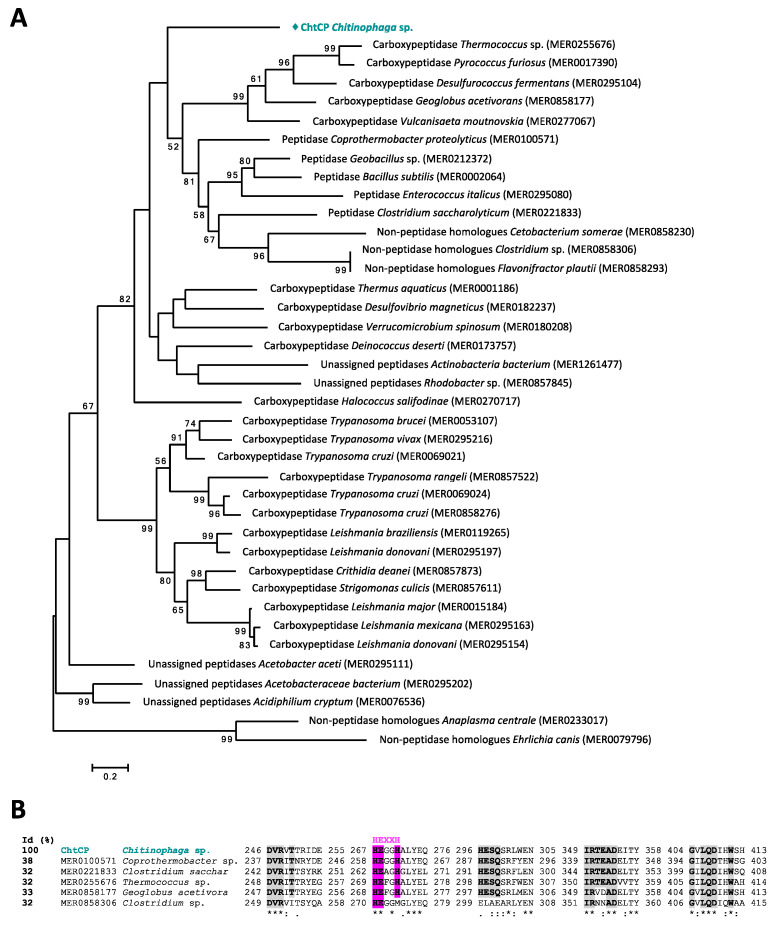
Sequence analysis of ChtCP. (**A**): Phylogenetic analysis by the Maximum Likelihood method. A total of 38 sequences of the M32 family were retrieved from the MEROPS database and together with ChtCP (in cyan), the evolutionary history was inferred by using the Maximum Likelihood method based on the JTT matrix-based model [51]. The tree with the highest log likelihood (-18173.3211) is shown here, and the percentage of trees in which the associated taxa clustered together is localized next to the branches. This tree is drawn to scale, with branch lengths measured in the number of substitutions per site. The sequence alignment was performed using ClustalW and the output file was used to perform the evolutionary analyses in MEGA7 [50]. The description of the sequence, microorganism of origin and MEROPS identity (between brackets) is shown for each sequence. (**B**): Sequence alignment of ChtCP and selected sequences from M32 family retrieved from the MEROPS database. The conserved motifs are highlighted in gray and the conserved motif of hyperthermophilic metallopeptidaes (HEXXH) in pink. The sequence alignment was built by using ClustalW and the sequence identity between ChtCP and other sequences is shown in the left hand side of the figure.

Through the automatic annotation tool RAST [64], 82 ORFs potentially encoding for peptidases were identified in the bacterial genome of *Chitinophaga* sp. Using a comparative search between different databases, a total of 62 peptidases belonging to 26 large groups of proteolytic enzymes (clans) were detected. In the current work, by exploring the previously generated datasets, we identified an ORF which could potentially codify a metallocarboxypeptidase with interesting physicochemical characteristics such as pH, temperature and thermostability. The translated protein of the selected ORF was denominated ChtCP. ChtCP consists of 1503 bp (% G + C = 32%) encoding a protein with 500 amino acids, at 57.38 kDa. The amino acid sequence of ChtCP was used for a homology comparison and the result showed that ChtCP shares 93% of its sequence identity with a not yet characterized M32 carboxypeptidase from *Chitinophaga jiangnisgensis* (WP073077496.1) (Table S1). By comparing a blastp with the PDB database, we observed that ChtCP showed a low structural similarity with other described carboxypeptidades, sharing 41.6% similarity with a thermostable carboxypeptidase from *Fervidobacterium islandicum* (PDB: 5E3X), 40.1% with a carboxypeptidase from *Thermus thermophilus* (PDB: 3HOA), 37.3% with a hydrolase from *Bacillus subtilis* (PDB: 3HQ2) and 37% with a carboxypeptidase from *Deinococcus radiodurans* (PDB: 5GIV) (Figure S1). In-silico analyzes, of sequence alignments and phylogenetic trees of ChtCP, revealed that ChtCP is a member of the carboxypeptidases M32 family. To infer the evolutionary history of ChtCP, 38 sequences of M32 family were retrieved from the MEROPS database and the tree was generated by the Maximum Likelihood method. Through phylogenetic analysis, we could observe that ChtCP is located in a separate branch but closer to a carboxypeptidase from *Thermococcus* sp. (MER0255676) (Figure 1A), a homologue of carboxypeptidase from *Thermus aquaticus* (Taq) and *Pyrococcus furiosus* (Pfu). Members of family M32 contain two zinc binding histidines and a catalytic glutamate in a zinc binding motif HEXXH (Figure 1B, with the conserved motif highlighted in pink). This family of peptidases are known for their high temperature stability [15] and for being widely distributed in several biological systems. The phylogenetic analyses revealed that ChtCP is related to the carboxypeptidases of the subfamily M32.001 and M32.002, which are known for their thermostability, a highly desirable characteristic for industrial applications. Other members of the M32 family are the enzymes TaqCP from *Thermus aquaticus*, Pfu from *Pirococcus furiosus* and BsuCP from *Bacillus subtilis*. The sequence alignment of ChtCP and selected members representing the M32 family showed the conserved motifs of hyperthermophilic metallopeptidases (HEXXH or HXXEX), which provides a signature for thermophilic metallopeptidases [32,33] (Figure 1B and Figure S1, in pink and blue), and also another 5 conserved domains (DXRXT, HPF, HESQ, IRXXAD and GXXQDXHW) involved in the binding or activity of metal ions and thermostable carboxypeptidase substrates [33] (Figure 1B and Figure S1, in gray). A comparison of the full-length amino acid sequence of ChtCP and its close homologues is shown in Figure S1. The in-silico analyzes of ChtCP revealed a molecular weight of 57.38 kDa, an extinction coefficient of 88.365, an isoelectric point of 5.61 and a theoretical melting temperature (Tm) above 65 °C; this information was used as a reference during the development of further experiments.

### 3.2. Expression and Evaluation of the ChtCP Quaternary Structure

The highest yield of soluble ChtCP protein was obtained by inducing the expression with 0.4 mM IPTG at 30 °C, 200 rpm, followed by 16 h of incubation after induction. The first stage of purification was performed using immobilized metal affinity chromatography, and it was possible to observe the partially purified ChtCP protein with a molecular mass of 57 kDa on the SDS-Page gel, as predicted by ProtParam. The second step of purification was performed by size exclusion chromatography, whose chromatographic profile shows the main elution of ChtCP around 78 mL column volume (Figure S2A), suggesting a molecular weight of approximately 74 kDa according to proteins tested in the same conditions (Figure S2B). Coupling the two purification steps, it was possible to obtain ChtCP with a high purity (Figure S2C). The pure protein was used for subsequent functional and crystallization assays. Our results indicate that ChtCP is likely to be a homo-dimer in its active form, a common structure for members from the M32 family of carboxypeptidases [65,66,67].

### 3.3. ChtCP, An Enzyme with a Wide Range of pH and Temperature

**Figure 2:**
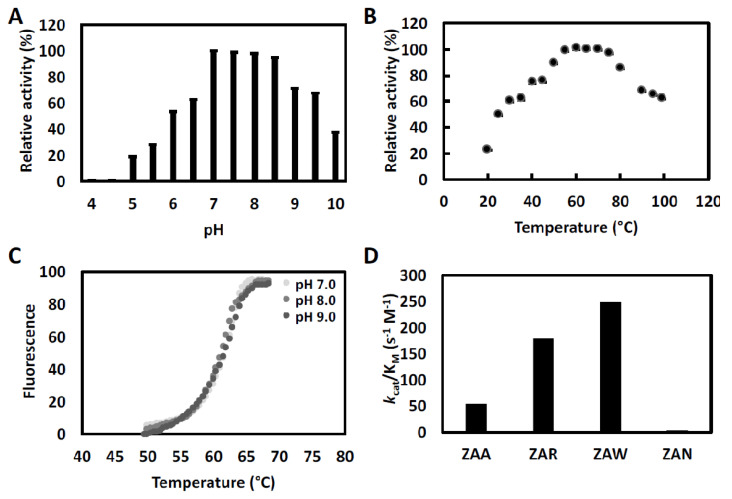
Functional characterization of ChtCP. (**A**): The pH effect on ChtCP activity. Buffers with different pH were tested: sodium citrate (pH 4.0, 4.5, 5.0, 5.5 and 6.0); tris-HCl (pH 7.0, 7.5, 8.0 and 8.5); sodium bicarbonate (pH 9.0, 9.5 and 10.0); (**B**): The temperature effect on ChtCP activity. A total of 1 mg of ChtCP in 100 mM tris-HCl pH 8.0 was incubated for 1 h at different temperatures and the residual activity measured after incubation with 2 mM ZAR. (**C**): melting temperature (Tm) to *ChtCP* determined via thermal shift assay, where their denaturation promote the release of Sypro orange and the fluorescence (λ_excitation_ = 410 nm, λ_emission_ = 610 nm) released is measured through a ramp of temperature from 40 to 80 °C. The experiment was performed in 50 mM tris-HCl buffer with different pH values (7.0, 8.0 and 9.0). Each condition was performed in triplicate and the data were further normalized. (**D**): Catalytic efficiency (*k*cat/K_M_) of ChtCP measured against benzyloxycarbonyl Ala-Arg (ZAR), benzyloxycarbonyl Ala-Ala (ZAA), benzyloxycarbonyl Ala-Trp (ZAW) or benzyloxycarbonyl Ala- Asn (ZAN). ChtCP at the concentrations of 0.41 to 1.6 µM were tested against the substrates at the concentration range from 0.195 to 25 mM. The reaction was performed in triplicate with 50 mM hepes and a pH of 8.0 at 65 °C.

ChtCP showed a wide activity range at different pH, from 4.0 to 10.5, and optimal pH values of between 7.0 to 9.0 (Figure 2A). For thermostability, the ChtCP protein showed relative activity above 80% between 55 to 80 °C, and activity above 60% at temperatures above 90 °C (Figure 2B), with the optimum temperature being observed within the range of 55 °C to 70 °C (Figure 2B). In contrast, the PfuCP enzyme [37] showed no activity at temperatures below 40 °C, under standard conditions. The reported carboxypeptidases from the bacterias *Sulfoloobus solfataricus* and *Thermus aquaticus* showed an optimal temperature between 85 °C and 80 °C, and PfuCP exhibits an optimal temperature above 90 °C [37,68,69] (Table S2). ChtCP had a theoretical melting temperature between 55 °C to 65 °C, and a melting temperature index (TI) of 1.73. Such values are very similar to the melting temperature (Tm) obtained in this study (61 °C). To investigate the melting temperature of ChtCP, the Tm was determined in the absence of metal ions and at different pH values (7.0, 8.0 and 9.0) and a similar Tm was observed for the three pH values tested (Figure 2C). ChtCP showed a high stability of its tertiary structure regardless of the pH (7.0, 8.0 and 9.0), while temperatures above 60 °C induced the denaturation of the α-helical structure of ChtCP, showing a transition to the irreversible state.

To investigate the sites P1’ and P1 of ChtCP at the C-terminal of peptide substrates, the specificity of the purified ChtCP substrate was examined by steady-state kinetics using synthetic substrates (ZAA, ZAR, ZAW and ZAN). ChtCP showed greater affinity for substrates with non-polar amino acids at the c-terminal (ZAW and ZAA) and the highest catalytic efficiency (*k*cat/K_M_) for polar-basic amino acids (Figure 2D, Table 1). The kinetic curves for each substrate can be observed in the Supplementary material, Figure S3.

### 3.4. The Effect of Metal Ions on Enzymatic Activity and on Temperature of Denaturation

**Figure 3:**
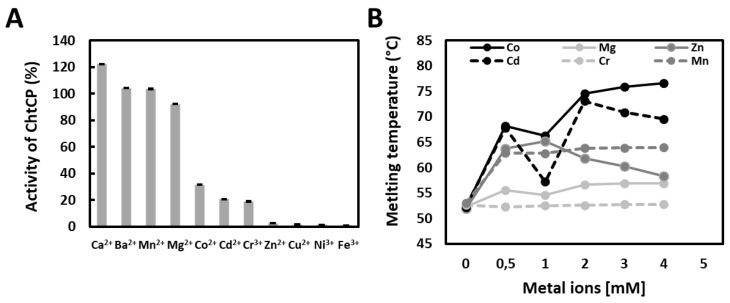
The effect of metal ions on ChtCP activity and melting temperature. (**A**): Effect of metal ions on ChtCP activity. The values were normalized to a control where no metal ions were added. (**B**): Melting temperature of ChtCP in the presence and absence of metal ions (Co^2+^, Cd^2+^, Mn^2+^, Mg^+2^, Cr^3+^ and Zn^2+^). 0.5 mM to 5 mM of CoCl_2,_ CdCl_2_, MnSO_4_, MgSO_4_, CrSO^4^ and ZnCl_2_) were added to 4 μg of purified ChtCP in hepes pH 8.0. The Tm was determined via thermal shift assay, being the released fluorescence of Sypro orange (λ_excitation_ = 410 nm, λ_emission_ = 610 nm) measured by qPCR Stratagene Mx 3000P (Agilent, Santa Clara, CA, USA) system in a ramp of temperature, from 50 °C to 80 °C.

Metalloenzymes catalyze a wide variety of reactions with high efficiency, selectivity and across a wide range of different conditions. These enzymes therefore combine the powerful reactivity of metal ions with the precise control of the electronic and steric properties provided by proteins [70]. The M32 family exhibits a dependence on metal ions, especially Zn^2+^. However, Zn^2+^ seems to have the opposite effect on ChtCP, resulting in a decrease of ChtCP activity by approximately 80% in its presence. A similar decrease in activity was observed for other cations such as Cr^3+^ and Cd^2+^. In contrast, ChtCP showed high dependence on Mn^2+^ and Co^2+^ ions. The activity of ChtCP increased by 2 and 2.5 times in the presence of these ions, respectively (Figure 3A). Such results are similar to those obtained by the keratin-degrading enzyme AW-1 of *Fervidobacterium islandicum* [32]. The results showed a strong stabilization of ChtCP by Co^2+^ (Figure 3B). When in the presence of 4 mM Co^2+^ the melting temperature of ChtCP increased by approximately 25.3 °C, from 52.5 °C to 77.8 °C (Figure 3B, black line). In contrast, the addition of Zn^2+^ and Cr^3+^ did not show as positive an effect on the stability of ChtCP. For example, when in the presence of 2 mM to 4 mM of these metals the melting temperature is around 60 °C and 53 °C (Figure 3B, gray line and light gray dotted line), 1.3 and 1.46 times lower, respectively, when compared to Tm in the presence of Co^2+^. Interestingly, the most pronounced variation in the Tm occurred when in the presence of metals at a concentration below 1 mM, for example, an increase of approximately 18 °C and 10 °C was observed when in the presence of Co^2+^ and Mn^2+^ (Figure 3B, black and dark gray dotted line), respectively, compared to the assays without the addition of metals. Except by the addition of Zn^2+^, we observed that in the presence of higher concentrations of binders the increase in Tm occurred more gradually and followed the expected trend for the metal binding domains [71].

### 3.5. Crystal Scructure of ChtCP

**Figure 4:**
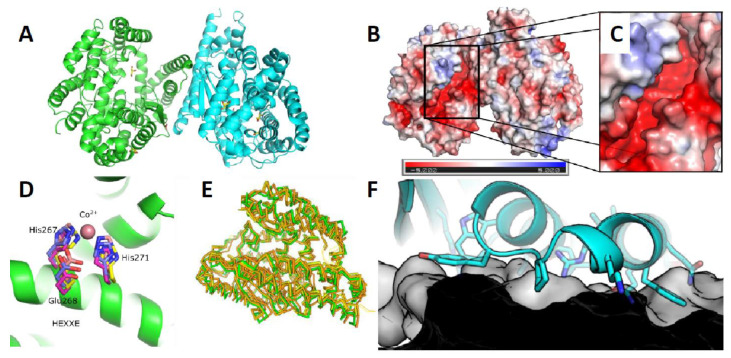
Crystal structure of ChtCP carboxypeptidase from *Chitinophaga* sp. (**A**): The crystal structure of the carboxypeptidase ChtCP. The protein forms a dimer in the crystals structure which is believed to be the active form. (**B**) The electrostatic potential surface calculated from the crystal structure of ChtCP. The surface is contoured from −5 to +5. (**C**): A zoomed in view of the negatively charged active site groove of ChtCP. (**D**): The highly conserved metal binding residues of ChtCP aligned to the closely related structures PDB: 3HOA and 3HQ2. (**E**): ChtCP aligned to the 5 most closely related protein structures in the PDB (5GIV, 5WVU, 1WGZ, 5WVU and 3HOA) calculated by the Dali server [72] (**F**): The flat interface dimer between the 2 monomers of ChtCP.

**Figure 5:**
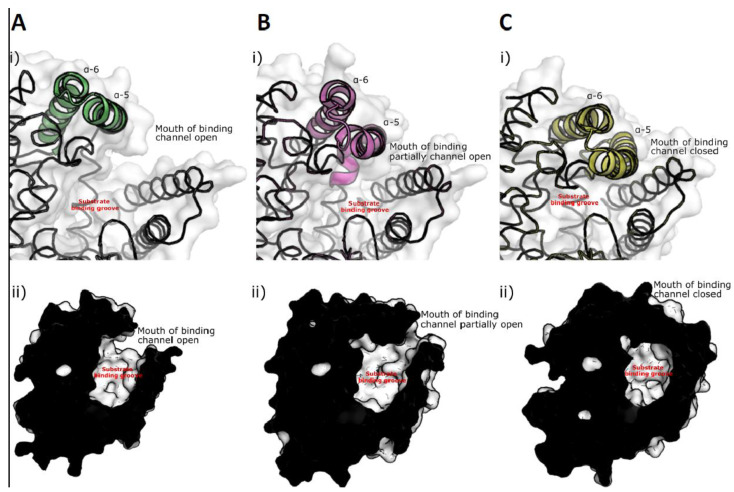
The opening of the mouth of the substrate binding channel in various structures of M32 Carboxypeptidases. (i) Shows the carton depiction of α-5 and α-6 (ii) shows a cross section through the surface representation. (**A**): PDB of ChtCP (PDB: 7A03), (**B**): PDB: 3HOA (r.m.s.d = 1.19 Angstrom), (**C**): PDB: 3HQ2 (r.m.s.d = 1.927 Angstrom).

The crystal structure of ChtCP was determined (Figure 4A) at a resolution of 1.39 Angstrom by molecular replacement in Phaser using the related structure PDB: 5GIV. Similar to other related proteins the structure of ChtCP is predominantly helical with one small 3 stranded β-sheet located close to the active site [38]. The structure can be divided into 2 subdomains one domain is associated with substrate binding whereas the other subdomain is associated with dimer formation. The substrate binding groove and active site is characterized by a deep predominantly negatively charged cleft (Figure 4B,C). This cleft contains the conserved HEXXH motif that is characteristic of M32 Carboxypeptidases. The HEXXH is similarly conserved in the new ChtCP structure with the 3 important residues aligning well with existing structures e.g., PDB: 3HOA and PDB: 3HQ2 in Figure 4D. However, no metals could be unambiguously assigned to be binding in the site of ChtCP site despite soaks with Zn^2+^, Co^2+^, Mg^2+^ and Mn^2+^ ions. ChtCP forms a dimer in solution and indeed this is observed in the crystal structure where the interface is formed by the stacking of two α-helices, α-1 and α-2 (Figure 4F). The interface is a broad flat interface not dominated by any deep binding clefts. However, the affinity of this interaction is not currently known. The overall structure shows very close similarity to other related structures with a RMSD of 1.37 to PDB: 5GIV which has 37% sequence identity (Figure 4E). However, there is some significant movement of several α helices across the various structures. The movement of these helices is associated with the opening and closing of the substrate binding groove. For example, in the structure of BsuCP (PDB: 3HQ2, Figure 5C), the substrate binding groove is closed by the movement of α-5 and α-6 [38]. However, in the structures of *Thermus thermophilus* M32 carboxypeptidase (PDB: 3HOA, Figure 5B) α-5 and α-6 are pushed out and the substrate binding groove is partially open [38]. In the structure presented here of ChtCP, the binding groove is in a very open conformation (Figure 5A) with α-5 and α-6 fully retracted. This is similar to the open conformation identified in the structure 5WVU [34]. This shows how the substrate would be able to enter the active site and confirms the full range of the mobility of this class of protein. This movement is surprising given the thermal stability of the protein, which one would have expected to be due to a rigid structure.

## 4. Discussion

The genome partial analysis of a gram-negative bacterium isolated from a bacterial consortium of sugarcane bagasse extracted from a bioethanol plant production, showed 75% similarity with the genome of the genus *Chitinophaga* sp. According to Kishi et al. 2017 [19], 62.4% of genes encoding proteins from this particular bacteria type were attributed to a putative function, while the rest were annotated as hypothetical proteins. By performing data mining and in-depth analysis of *Chitinophaga* sp., 72 ORFs were annotated as potential genes for encoding proteolytic enzymes. From these identified genes, the *chtcp* gene was selected due to the great potential shown in the sequence analysis and the structure prediction for the codified protein ChtCP. The sequence analysis of ChtCP revealed that this protein is a member of the carboxypeptidase family M32. This means ChtCP is potentially a novel thermostable carboxypeptidase with interesting properties similar to the enzymes from *Thermus aquaticus* and *Pirococcus furiosus* (Figure 1 and Figure S1). The functional characterization of ChtCP protein showed an optimum pH of 7.0, with the enzymatic activity reduced by 40% and 30% at a pH below 6.5 or above 9.0, respectively. As a comparison, the PfuCP enzyme showed an optimum pH at 6.2 and 6.6 [37], FisCP showed an optimum at pH 7.0 [32], and TcrCP also showed its highest activity at pH 6.2. The decrease in activity of ChtCP outside the pH range of 7.0 to 9.0 is mainly due to irreversible changes in the protein structure caused by the pH. Nevertheless, because ChtCP is active over a wide range of pH and temperature and particularly has increased activity at temperatures above 40 °C, understanding this enzyme is of great value for many biotechnological applications. In addition, the fact that ChtCP is more active at higher temperatures favors the increase in the solubility of the substrate and the product released [10,73]. Also, it should be noted that the conformational movements that are important for the activity of the enzymes require thermal energy. In other words, the flexibility and function of a given enzyme is inevitably coupled with temperature. For example, if the environment is very cold, the enzyme can get stuck in an inactive conformation [74]. This is not surprising, since enzymes change their conformations during catalysis and, therefore, can exist as a set of different conformations depending on their stability and function. Indeed, when the structure of ChtCP presented in this study is compared to related protein structures a conformational change that leads to the opening and closing of the substrate binding cleft can be seen across the structures (Figure 5). The structure of ChtCP as well as giving insights into the potential opening and closing of the substrate cleft allows an understanding of the potential substrate tolerance of ChtCP. This is of particular importance when selecting possible enzymes for new applications and would allow the rational alteration of ChtCP to optimize it for new processes. However, more detailed studies would be required to form reliable conclusions related to substrate selectivity. In addition to ChtCP being expressed in soluble form and being active over a wide range of pH and temperatures, ChtCP also presented a catalytic efficiency (*k*_cat_/k_M_) of 1.79 × 10^2^ and 2.48 × 10^2^ when tested against ZAR and ZAW, respectively. The higher enzymatic activity of ChtCP may be correlated with the presence of two catalytic motifs [33] _76_HXXEX_80_ (_76_HHPEL_80_, Figure S1, in light blue highlight) and _267_HEXXH_271_ (Figure S1, in pink highlight). The increase in the reaction rate of ChtCP in the presence of Co^2+^ and Mn^2+^ is due to the electronic nature of the metals, which activates the water molecules, allowing the formation of the intermediate state (enzyme-substrate), which consequently increases the speed of the reaction [10]. No activity of ChtCP was detected when the enzyme was tested in the presence of EDTA, indicating that the enzyme activity is dependent on metallic cations.

Cofactor binding is of paramount importance in vivo, with the loss of the coenzyme causing total loss of enzyme activity. The metal which is physiologically relevant for M32 family is uncertain, as illustrated by the variety of metallic cations (Zn^2+^ or Co^2+^) found in the active site of the M32 superfamily of carboxypeptidases [38,65,66]. This scarcity of information on the preference of cations makes it difficult to discern whether the selection of the cation reflects a preference of the proteins or a greater availability of a specific metal in the cellular environment [75]. In this context, due to the fact that ChtCP showed a similar affinity between Co^2+^ and Mn^2+^ and is active in the presence of other metals (Mn^2+^, Mg^2+^, Co^2+^, Cd^2+^, Cr^3+^ and Zn^2+^), these features together make ChtCP very attractive in terms of its physiology when compared to other homologous proteins from M32 family, which are usually only active in the presence of Zn^2+^ or Co^2+^. The wide activity of ChtCP for different metal ions also makes this protein interesting for applications in an intracellular environment, due to the great variety of metals and different concentrations that the intracellular environment demands. In this work, we have shown the functional and structural characterization of ChtCP, a new member of the M32 family, and the first metallopeptidase described to show the activity in the presence of Mn^2+^.

## Figures and Tables

**Table 1:** Functional characterization of ChtCP versus substrates with benziloxicarbonil (Z).

Substrate	Abbreviation	*k*_cat_ (s^−1^)	k_M_ (M)	*k*_cat_/k_M_ (s^−1^·M^−1^)
Z-Ala-Ala-OH	ZAA	1.55	2.81 × 10^−2^	5.53 × 10^1^
Z-Ala-Arg-OH	ZAR	6.28 × 10^−1^	3.51 × 10^−3^	1.79 × 10^2^
Z-Ala-Trp-OH	ZAW	2.72 × 10^−1^	1.10 × 10^−3^	2.48 × 10^2^
Z-Ala-Asn-OH	ZAN	1.48 × 10^−-1^	3.41 × 10^−2^	4.34

## Data Availability

The data presented in this study are available on request from the corresponding authors.

## Supplementary Materials

The following are available online at https://www.mdpi.com/2076-2607/9/2/393/s1, Table S1. Sequence comparison of ChtCP against NCBI database, Figure S1. Amino acid sequence comparison of the ChtCP and homologues, Table S2. Functional and kinetic profile summary of ChtCP and another carboxypeptidases representing six M32 subfamilies described in the literature, Figure S2. Superexpression and purification of ChtCP in its active form, Table S3. Data collection and refinement statistics of ChtCP, Figure S3. Effect of substrate concentration on ChtCP initial rate.
