# Supplementary material for: From Data Mining of Chitinophaga sp. Genome to Enzyme Discovery of a Hyperthermophilic Metallocarboxypeptidase
Source: Microorganisms. 2021 Feb 14;9(2):393. doi: 10.3390/microorganisms9020393 (PMC7918520; doi:10.3390/microorganisms9020393)
Supplement: Supplementary file 1 [file microorganisms-09-00393-s001.pdf]

From data mining of *Chitinophaga* sp. genome to enzyme discovery of a hyperthermophilic metallocarboxypeptidase

Gabriela Cabral Fernandes[1,2], Elwi Guillermo Machado Sierra[1,3], Paul Brear[4], Mariana Rangel Pereira[4,5*] and Eliana G. M. Lemos[1,*]

[1]Department of Technology, São Paulo State University (UNESP), Jaboticabal, São Paulo State, post code 14884-900, Brazil; gfernandes1403@gmail.com (G.C.F); egerle@fcav.unesp.br (E.G.M.L)

[2]Graduate Program in Agricultural and Livestock Microbiology, São Paulo State University (UNESP), School of Agricultural and Veterinarian Sciences, Jaboticabal, São Paulo State, post code 14884-900, Brazil;

[3]Laboratorio de Investigación en Microbiología, Facultad de Ciencias Básicas y Biomédicas, Universidad Simón Bolívar, Barranquilla post code 080002, Colombia; elwi.machado@unisimonbolivar.edu.co (E.G.M.S)

[4]Department of Biochemistry, University of Cambridge, Cambridge, post code CB2 1GA, United Kingdom; mr629@cam.ac.uk (M.R.P); pdb47@cam.ac.uk (P.B)

[5]CAPES Foundation, Ministry of Education of Brazil, Brasília - DF, post code 70.040-02, Brazil

[*]Corresponding author:
Eliana G. M. Lemos: egerle@fcav.unesp.br; elianag.lemos@gmail.com
Mariana Rangel Pereira: mr629@cam.ac.uk; mrangel8@hotmail.com

Table S1. Sequence comparison of ChtCP against NCBI database.

Description	Microorganism	Query cover (%)	Identity (%)	NBCI access code
Carboxypeptidase M32	*Chitinophaga jiangningensis*	99	93	WP073077496.1
Carboxypeptidase M32	*Chitinophaga dinghuensis*	99	88	WP111591735
Carboxypeptidase M32	*Chitinophaga vietnamensis*	99	81	WP143305023
Carboxypeptidase M32	*Chitinophaga arvensicola*	99	80	WP089893195.1
Carboxypeptidase M32	*Chitinophaga* sp.	99	78	WP160711557

```
           ....|....| ....|....| ....|....| ....|....| ....|....| ....|....| ....|....| ....|....| ....|....| ....|....|
               5         15         25         35         45         55         65         75         85         95
ChtCP      MPGSKSTAEQ YAAYKQKMQK IADVRNAIAV LGWDQETYLP EKGAGFRGQQ ITTLSTIAHE LFTAPELGSL LHELHH---H PELDAVQQKN IALSLEDYDK
5E3X       ---------- MEELKSYYKR VAKYYSAAAL LYWDMQTYMP KDAGPYRAEV LSEIGTYAFK QITDDALGKL LETAQP---Q SEID---EKL VYVGKKEYYK
3HOA       -MTP---EAA YQNLLEFQRE TAYLASLGAL AAWDQRTMIP KKGHEHRARQ MAALARLLHQ RMTDPRIGEW LEKVEGSPLV QDPLSDAAVN VREWRQAYER
3HQ2       ----MEIHTY EKEFFDLLKR ISHYSEAVAL MHWDSRTGAP KNGSEDRAES IGQLSTDIFN IQTSDRMKEL IDVLYER--F DDLSEDTKKA VELAKKEYEE
5GIV       -MTTTRQDTQ WQQLTEHWQE LADFGGIEAL LGWDQSTFLP AGAAEDRARQ QSLLAGLRHA RATDAGYGKL LDAASSR--- SDLSPEQARM VQVARQDFEK
                           .  :.  :      *:   **  *  *   .   *..     :.   .    *     .  :.          :         :    : : .

           ....|....| ....|....| ....|....| ....|....| ....|....| ....|....| ....|....| ....|....| ....|....| ....|....|
             105        115        125        135        145        155        165        175        185        195
ChtCP      NKKYPASLVA EISEATNQAY HAWIKARKAN DYQVFEPALA RMVELKR--- ------KETT VLGYEDHPYN ALLNEYEKGA NVDMLDTIFT EVKTALSPLL
5E3X       YKKVPPELFQ EIMITSTMLE QKWEIAKPRG DFEEVRPLLE KIVDLSR--- ------KYAD ILGYEGEPYN ALLDLYEPGM KAEEVDQIFS KVRDFIVEVL
3HOA       ARAIPERLAV ELAQAESEAE SFWEEARPRD DWRGFLPYLK RVYALTKEKA EVLFALPPAP GDPPYGELYD ALLDGYEPGM RARELLPLFA ELKEGLKGLL
3HQ2       NKKIPEAEYK EYVILCSKAE TAWEEAKGKS DFSLFSPYLE QLIEFNK--- ------RFIT YWGYQEHPYD ALLDLFEPGV TVKVLDQLFA ELKEAIIPLV
5GIV       ATRIPAEFVR EFSGHVGQSY SAWTEARPAN DFGRMVPYLE KTLDLS---- -----LQAAS YFPEFGDPLD YYINESDEGM TAEQVGQVFA ELRAALVPLA
               *      *            *  *:  . *:  . * *  :   :.                      .  :   ::  : *   .  :  :*: :::  :  :

           ....|....| ....|....| ....|....| ....|....| ....|....| ....|....| ....|....| ....|....| ....|....| ....|....|
             205        215        225        235        245        255        265        275        285        295
ChtCP      DDIAKQTPAR RD-FLHLHFD RDKQWQLGID LLRQMAYDMS AGRQDISEHP FTTSFNPLDV RVTTRIDEND FSNMTWSCIH EGGHALYEQG LPTEQYGLPC
5E3X       EKIER-LPKS ED-PFNREIG VDKQKEFSNW LLHYLKYDFT KGRLDVSAHP FTNPIGLNDV RITTRYIVND IRNSIYSTIH EFGHALYALS IPTEFYGLPI
3HOA       DRILGSGKRP DTSILHRPYP VEAQRRFALE LLSACGYDLE AGRLDPTAHP FEIAIGPGDV RITTRYYEDF FNAGIFGTLH EMGHALYEQG LPKEHWGTPR
3HQ2       KQVTASGNKP DTSFITKAFP KEKQKELSLY FLQELGYDFD GGRLDETVHP FATTLNRGDV RVTTRYDEKD FRTAIFGTIH ECGHAIYEQN IDEALSGTNL
5GIV       DAVIAAG-AP RTDFLGRGFA QERQLAFGER VIRDYGYDFR RGRQDLTHHP FMTRLGGHDV RITTRVKEQD PTDALYSTLH EAGHALYEQG VDAAFLGTPL
           . :           :       : *  :.  .:    **:   ** * : ** *   :.   ** *:***   .        :. :* * ***:*  . :      *
                                                                                                     ▲ ▲  ▲

           ....|....| ....|....| ....|....| ....|....| ....|....| ....|....| ....|....| ....|....| ....|....| ....|....|
             305        315        325        335        345        355        365        375        385        395
ChtCP      GEAASLGIHE SQSRLWENNV GRSLNFWKFQ YPRIQALFPE QLGNVSLQEF YKAINHVQPS LIRTEADEIT YHFHIMIRYE IEKGLIDGSI STKDLNKTWN
5E3X       GSSASYGFDE SQSRFWENVV GRSLAFWKGI YSKFIEIVPE MRG-YSVEEL WRAVNRVQRS FIRTEADEVT YNLHIIIRFE IERELINGEL SVKDVPDKWN
3HOA       GDAVSLGVHE SQSRTWENLV GRSLGFWERF FPRAREVFAS -LGDVSLEDF HFAVNAVEPS LIRVEADEVT YNLHILVRLE LELALFRGEL SPEDLPEAWA
3HQ2       SDGASMGIHE SQSLFYENFI GRNKHFWTPY YKKIQEASPV QFKDISLDDF VRAINESKPS FIRVEADELT YPLHIIIRYE IEKAIFSNEV SVEDLPSLWN
5GIV       GGGVSAGVHE SQSRLWENLV GRSRAFWAAY FGDWRDTFPE QLAGVTEEEM YRAVNTVSRS LIRTDADELT YNLHVITRFE LEREMLAGKL AVRDLADAWH
           . ..* *..* ***  :** : **.  **   :        .        : :::   *:*  . * :**.:***:* * :*:: * * :*  :: ..: : .*: . *
                    ▲

           ....|....| ....|....| ....|....| ....|....| ....|....| ....|....| ....|....| ....|....| ....|....| ....|....|
             405        415        425        435        445        455        465        475        485        495
ChtCP      DYYRQYLHVE VPNDTQGVLQ DIHWSHGSFG -YFPTYSLGS FYAAQFFTTA QKQVPDLDVS IASGNYQPLL EWLRNNIHPF GRFYTSNELC QKITGNPLQF
5E3X       ELYKKYLGLD VPNNTLGCMQ DPHWFGGNFG -YFPTYALGN LYAAQIFEKL KEEIN-FEEV VSAGNFEIIK NFLKEKIHSK GKMYEPSDLI KIVTGKPLSY
3HOA       EKYRDHLGVA PKDYKDGVMQ DVHWAGGLFG -YFPTYTLGN LYAAQFFQKA EAELGPLEPR FARGEFQPFL DWTRARIHAE GSRFRPRVLV ERVTGEAPSA
3HQ2       QKYQDYLGIT PQTDAEGILQ DVHWAGGDFG -YFPSYALGY MYAAQLKQKM LEDLPEFDAL LERGEFHPIK QWLTEKVHIH GKRKKPLDII KDATGEELNV
5GIV       AAYEQNLGLR APSDVDGALQ DVHWYFGPIG GSFQGYTIGN VLSAQFYAAA EAANPGLEAD FARKDFSRLH GWLRENVYRH GRRWTPGELI ERATGQALTA
             *.. * :        * :* * ** *  :*  *  *::*  . :**:               ::  .    ::  :   :   .::  *    .  :  :   **:

           ....|....| ....|....
             505        515
ChtCP      SYFLDYAAGK FLRG-----
5E3X       ESFVRYIKDK YSKVYEIEL
3HOA       RPFLAYLEKK YAALY----
3HQ2       RYLIDYLSNK YSNLYLL--
5GIV       GPYLKYLRGK YGELYGV--
              : *   * :
```

Figure S1. Amino acid sequence comparison of the ChtCP and homologues. The identical amino acids are shown by asterisks below the sequence. The conserved domains are shown in grey and the highly conserved motifs of M32 family in light blue (HXXEX) and in pink (HEXXH). The catalytic residues are indicated with a triangle (▲). The PDB codes correspond to *Fervidobacterium islandicum* (PDB: 5E3X), *Thermus thermophilus* (PDB: 3HOA), *Bacillus subtilis* (PDB: 3HQ2) and *Deinococcus radiodurans* (PDB: 5GIV).

Table S2. Functional and kinetic profile summary of ChtCP and another carboxypeptidases representing six M32 subfamilies described in the literature. The cofactor presented here refer to the assays where it was observed an increase in enzyme activity when in the presence of mentioned metal; and (*) refers to enzymes extremely depend of a specific metal. NA is the abbreviation of not applicable; and Cbz, Z, Fa are the abbreviation of carbobenzoxy, benziloxicarbonil and Fa: N-(3-[2-furyl]acryloyl), respectively.

**Enzyme**	**Affinity for aa in c-terminus**	**Optimum T (°C)**	**Optimum pH**	**Cofactor**	**K_M (M) [substrate]**	**k_{cat} (s^{-1})**	**k_{cat} / K_M (s^{-1} M^{-1})**	**Reference**
ChtCP	Nonpolar	55 to 75	7 to 8.5	Co^{+2} - Mn^{+2}	3.51 E-03 [Z-Ala-Arg]	6.28E-01	1.79E+02	This work
Carboxipeptidase Taq - TaqCP (M32.001)	Aromatic polar and neutral	75 to 80	7.5 to 8.0	Co^{+2}*	7 E-04 [Cbz-Phe-Tyr]	NA	NA	[35] [69]
Carboxypeptidase Pfu - PfuCP (M32.002)	Polar, Basic or neutral, Aromatic	90 to 100	6.2 to 6.5	Co^{+2} - Zn^{+2}	9 E-04 [Z-Ala-Arg]	6 E+02	6.6E+05	[37]
Carboxypeptidase TcMCP-1 (M32.003)	Basic	37	6.2	Co^{+2}	1.69 E-04 [Fa-Ala-Lys]	5.7	3.3E+04	[67]
Carboxypeptidase TcMCP-2 (M32.004)	Aromatic and aliphatic	37	7,6	Co^{+2}	3.4 E-05 [Fa-Phe-Phe]	11	3.2E+05	
Carboxypeptidase LmaCP1 (M32.005)	wide variability	37	7.5	Co^{+2}	7 E-03 [Z-Ala-Arg]	11.9	1.7E+03	[38]
Carboxypeptidase *Bsu*CP (M32.006)	Basic and aromatic	70	7.5	Mn^{+2} - Zn^{+2}	2.8 E-04 [Z-Ala-Arg]	65.8	2.3E+05	[38]

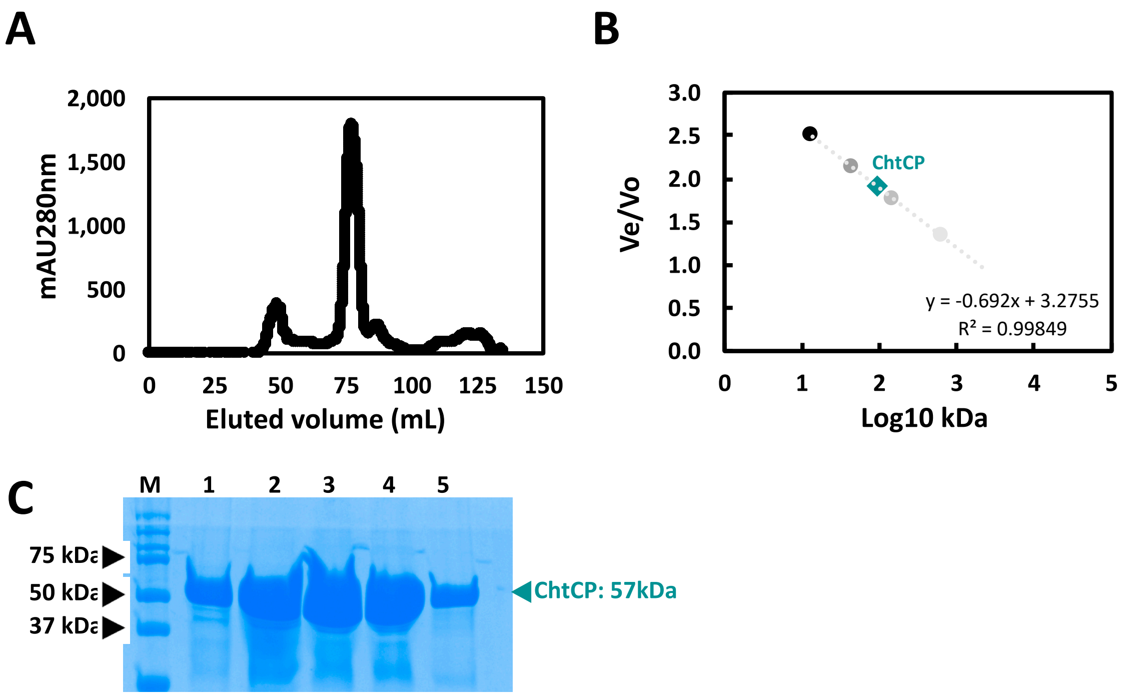

Figure S2. Overexpression and purification of ChtCP in its active form. A- ChtCP elution profile by size exclusion using superdex 16/60 200. Black line corresponds to mAU measured at 280 nm, and peak corresponds to fractions with high enzymatic activity. B- Analysis of the molecular weight of native ChtCP (◆) with standard proteins [Bovine thyroglobulin (670 kDa), γ-globulin (150 kDa), albumin (43 kDa), Ribonuclease A (13.7 kDa) and ρ-amino acid benzoic (pABA) (0.13 kDa)] using linear regression analysis to determine the molecular weight of the homodimer. C- SDS-PAGE analysis of the fractions obtained by gel filtration chromatography, being M: molecular weight marker, 1 to 5: eluted fractions from size exclusion, and the cyan arrow (◄) indication the protein band of ChtCP

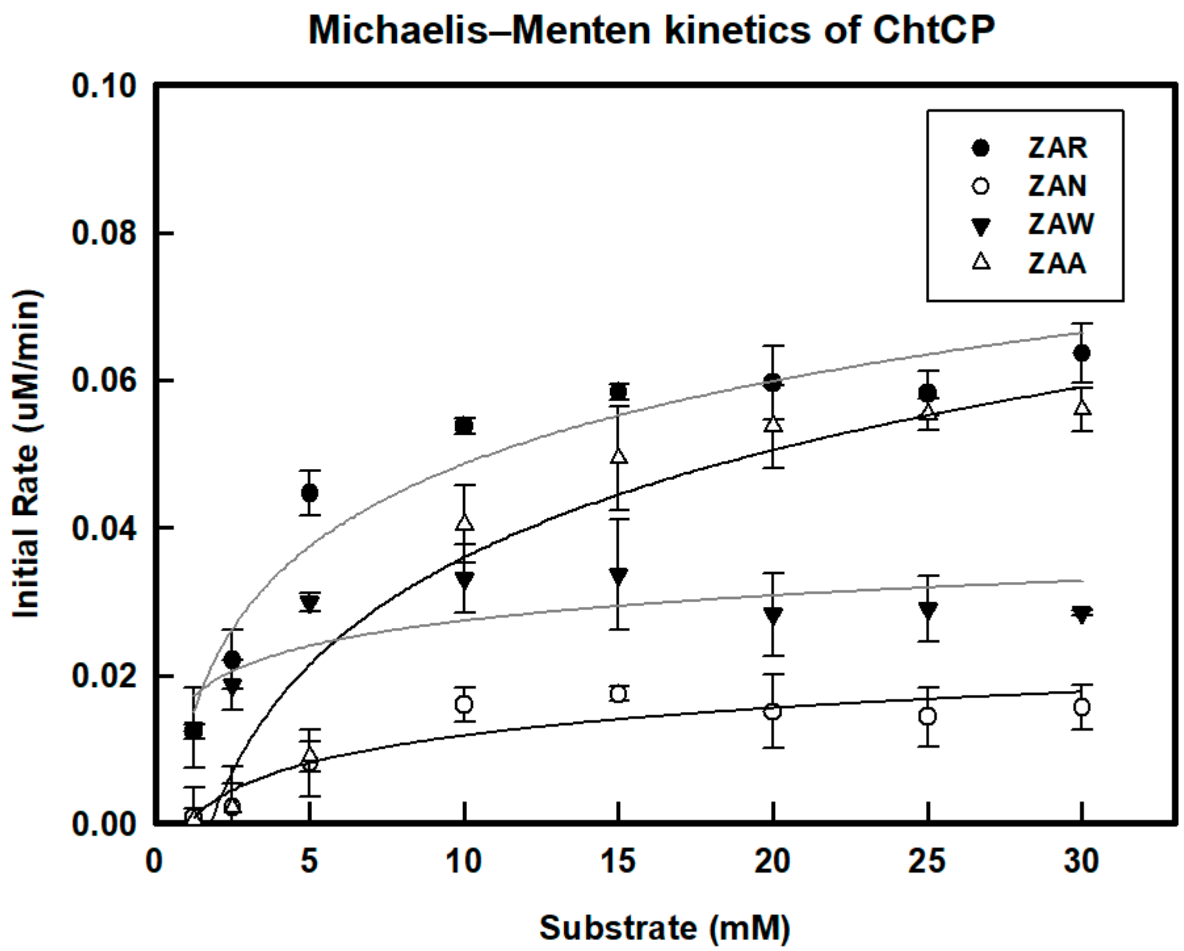

Figure S3. Effect of substrate concentration on ChtCP initial rate. The obtained data of ChtCP could be fitted to the model of Michaelis-Menten kinetics, and the non-linear regression of initial velocity versus substrate concentration is shown here. The activity of ChtCP was measured against benzyloxycarbonyl Ala-Arg (ZAR), benzyloxycarbonyl Ala-Ala (ZAA), benzyloxycarbonyl Ala-Trp (ZAW), benzyloxycarbonyl Ala- Asn (ZAN) or benzyloxycarbonyl Ala-Asp (ZAD) at the concentration range from 0.195 to 25 mM. The reaction was performed in triplicate in 50 mM hepes pH 8.0 at 65 °C.

Table S3. Data collection and refinement statistics of ChtCP.

Contents	ChtCP
Data collection	
PDB code	7A03
Data collection date	07/08/2017
Beamline	Diamond beamine IO4
Wavelength	0.9795
Resolution range	50.59 - 1.39 (1.43 - 1.39)
Space group	P 21 21 21
Unit cell	93.10 110.27 113.87 90 90 90
Total reflections	2347666 (171612)
Unique reflections	234729 (17198)
Multiplicity	10.0 (10.0)
Completeness (%)	100.0 (100.0)
Mean I/sigma(I)	13.4 (1.1)
Wilson B-factor	14.63
R-merge	0.077 (1.689)
R-meas	0.086 (1.882)
R-pim	0.038 (0.821)
CC1/2	0.999 (0.576)
Refinement statistics	
Resolution range	50.59 - 1.39 (1.44 - 1.39)
Reflections used in refinement	234589 (21452)
Reflections used for R-free	11363 (1025)
R-work	0.25 (0.43)
R-free	0.27 (0.42)
CC(work)	0.943 (0.615)
CC(free)	0.933 (0.599)
Number of non-hydrogen atoms	8775
macromolecules	8139
ligands	52
solvent	584
Protein residues	987
R.m.s deviations	
RMS(bonds)	0.006
RMS(angles)	0.84
Ramachandran distribution	
Ramachandran favored (%)	97.76
Ramachandran allowed (%)	1.93
Ramachandran outliers (%)	0.31
Rotamer outliers (%)	0
Clashscore	2.55

Average B-factor	22.15
macromolecules	21.73
ligands	31.19
solvent	27.21
